# Evaluation of anorectal function using real-time tissue elastography before and after preoperative chemoradiotherapy

**DOI:** 10.1007/s00384-024-04633-8

**Published:** 2024-04-25

**Authors:** Akira Sakamoto, Kazuhito Sasaki, Hiroaki Nozawa, Koji Murono, Shigenobu Emoto, Yuichiro Yokoyama, Hiroyuki Matsuzaki, Yuzo Nagai, Shinya Abe, Takahide Shinagawa, Hirofumi Sonoda, Soichiro Ishihara

**Affiliations:** Department of Surgical Oncology, Faculty of Medicine, The University of Tokyo, 7-3-1 Hongo, Bunkyo-Ku, Tokyo, 113-8655 Japan

**Keywords:** Chemoradiotherapy, Anorectal function, Internal anal sphincter, Sclerosis, Elasticity

## Abstract

**Purpose:**

This study aimed to clarify the relationship between changes in elasticity and anorectal function before and after chemoradiotherapy.

**Methods:**

This is a single-center prospective cohort study (Department of Surgical Oncology, The University of Tokyo). We established a technique to quantify internal anal sphincter hardness as elasticity using transanal ultrasonography with real-time tissue elastography. Twenty-seven patients with post-chemoradiotherapy rectal cancer during 2019–2022 were included. Real-time tissue elastography with transanal ultrasonography was performed before and after chemoradiotherapy to measure internal anal sphincter hardness as “elasticity” (hardest (0) to softest (255); decreased elasticity indicated sclerotic changes). The relationship between the increase or decrease in elasticity pre- and post-chemoradiotherapy and the maximum resting pressure, maximum squeeze pressure, and Wexner score were the outcome measures.

**Results:**

A decrease in elasticity was observed in 16/27 (59.3%) patients after chemoradiotherapy. Patients with and without elasticity decrease after chemoradiotherapy comprised the internal anal sphincter sclerosis and non-sclerosis groups, respectively. The maximum resting pressure post-chemoradiotherapy was significantly high in the internal anal sphincter sclerosis group (63.0 mmHg vs. 47.0 mmHg), and a majority had a worsening Wexner score (60.0% vs. 18.2%) compared with that of the non-sclerosis group. Decreasing elasticity (internal anal sphincter sclerosis) correlated with a higher maximum resting pressure (*r* = 0.36); no correlation was observed between the degree of elasticity change and maximum squeeze pressure.

**Conclusion:**

Internal anal sphincter sclerosis due to chemoradiotherapy may correlate to anorectal dysfunction.

**Supplementary Information:**

The online version contains supplementary material available at 10.1007/s00384-024-04633-8.

## Introduction

Currently, preoperative chemoradiotherapy (CRT) is the standard, global conventional treatment for lower-advanced rectal cancer to suppress local recurrence and improve the rate of anal preservation under tumor regression. Alternatively, total neoadjuvant therapy is gaining attention owing to its advantages, including improved chemotherapy completion rates, preoperative downstaging, and early initiation of systemic treatment for micrometastases [1–4].

A few studies suggest that preoperative CRT affects the development of anorectal dysfunction, low anterior resection syndrome, and fecal incontinence after rectal surgery [5–7]. However, others indicate that it has no effect; thus, the effects of CRT remain debatable [8, 9]. Furthermore, no definitive conclusion has been reached for the short-term effects of preoperative CRT on anal function [10, 11]. Pathologic features of irradiated healthy tissues include progressive fibrosis. Furthermore, a report on the internal anal sphincter (IAS) of surgical specimens from patients who underwent rectal dissection after preoperative CRT revealed a greater fibrosis tendency than in those who did not undergo preoperative CRT [12]. Hence, we speculated that CRT-induced fibrosis may induce IAS sclerosis, which may consequently be associated with postoperative anal dysfunction. Therefore, in 2021, Fukui established a method to quantify IAS hardness and defined it as elasticity using real-time tissue elastography (RTE) on the endoanal ultrasonography (EAUS) [13]. Santoro et al. have already reported that endorectal ultrasonography was useful in assessing the depth of submucosal invasion in early rectal cancer [14], but there are no reports of its use in functional assessment so far. Hence, we aimed to determine the relationship between changes in elasticity, anorectal function, and Wexner score before and after CRT.

## Materials and methods

### Patients

**Fig. 1:**
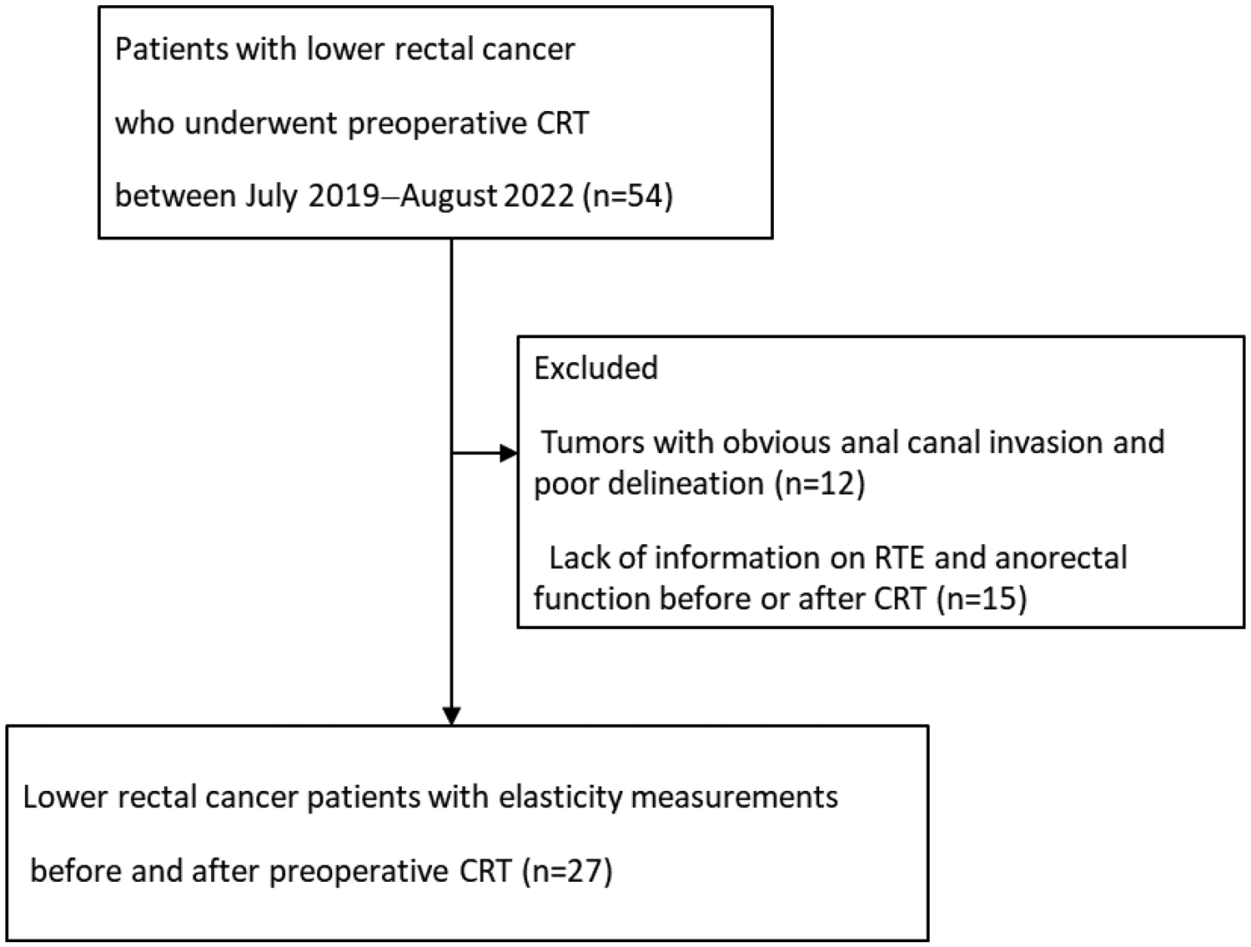
Flow diagram of the study. Patients with rectal cancer invading the anal canal and those who did not undergo real-time tissue elastography before or after chemoradiotherapy were excluded. CRT, chemoradiotherapy; RTE, real-time tissue elastography

We prospectively enrolled 27 patients with locally advanced rectal cancer who underwent CRT prior to rectal surgery between June 2019 and August 2022 at the Department of Surgical Oncology, The University of Tokyo. Patients with rectal cancer invading the anal canal and those who did not undergo RTE before or after CRT were excluded (Fig. 1).

The indication for CRT was primary adenocarcinoma of the lower rectum below the middle Houston valve without distant metastasis (cT3–cT4, any N, M0). Staging was based on the American Joint Committee on Cancer staging system, 8th edition [15]. The electronic medical record was accessed to retrieve clinical information on the patient background, including age, sex, tumor location from the anal verge before CRT, cStage, chemotherapy regimen, and tumor regression grade. The Japanese Classification of Colorectal Carcinoma was used to determine tumor regression grade [16].

For preoperative CRT, radiation therapy was initiated on the first day of chemotherapy and administered at 1.8 Gy/day five times weekly for a total dose of 50.4 Gy. On days 1–5, 8–12, 15–19, 22–26, and 29–33, patients were administered 5-fluorouracil (5-FU)-based chemotherapy with tegafur/uracil (300 mg/m^2^/day) and leucovorin (75 mg/body weight/day) orally thrice daily. Subsequently, the patients were treated with irinotecan intravenous infusion.

Furthermore, IAS elasticity before and after CRT, anorectal function test values (MRP; maximum resting pressure, MSP; maximum squeeze pressure), and Wexner score were examined. Patients with and without elasticity decrease before and after CRT comprised the IAS sclerosis and non-sclerosis groups, respectively. The correlation between both groups and the worsening of MRP, MSP, and Wexner score before and after CRT was investigated. Similarly, the correlation between the changes in elasticity before and after CRT and anorectal function test values was examined.

## Ethics approval

The University of Tokyo Ethics Committee approved the study protocol (No. 10046-(5) and No. 3252-(16)). Written informed consent was obtained from all patients. The study has been reported consistent with the Strengthening the Reporting of Observational Studies in Epidemiology (STROBE) guideline [17].

### Real-time tissue elastography

**Fig. 2:**
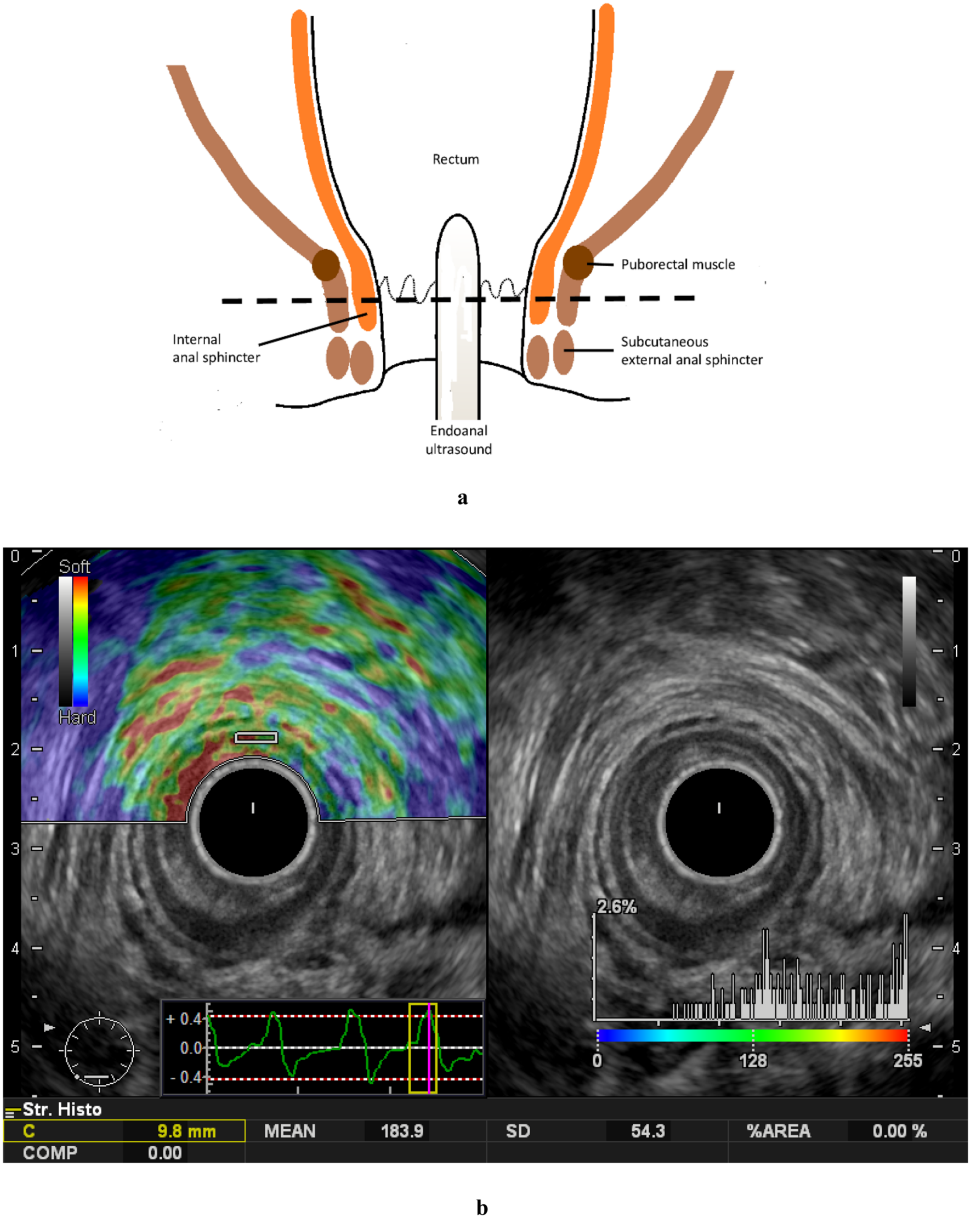
**a** Schematic representation of the anal canal and ultrasonography images. **b** Images of real-time tissue elastography performed at the level of anal canal between the puborectalis muscle and the subcutaneous external anal sphincter. The B-mode ultrasound and elastography images are on the right and left, respectively. In the right frame, intra-anal and B-mode ultrasound images displaying the internal anal sphincter (white arrow) and external anal sphincter (yellow arrow). A region of interest was established in the internal anal sphincter, and elasticity was measured

B-mode ultrasound images of the anal canal and RTE were performed using a Noblus ultrasound system (Hitachi, Tokyo, Japan) equipped with a 10-MHz rectal probe (EUP-R54AW-19; Hitachi, Tokyo, Japan). Two surgeons performed the ultrasonography, including RTE. All patients were placed in the left lateral position, and the rectal probe was inserted into the anal canal for measurements (Fig. 2a). RTE was performed on each patient with freehand manual compression in the anterior, left, posterior, and right positions to confirm that the IAS was well-detected and its elasticity could be assessed. Fukui et al. RTE method was followed [13], and IAS elasticity—the stiffness of the IAS at 6 o'clock of the anus—was recorded and evaluated (hardest (0) to softest (255), decrease in elasticity means sclerotic change). Figure 2b presents posterior IAS image evaluation.

### Anorectal and bowel function

Each anorectal functions of the patients, such as MRP and MSP, were measured by employing the rapid pull-through method using a one-channel a catheter for pressure recording and a computer system (GMMS-100R-SI instrument, Star Medical, Japan). Subsequently, IAS elasticity was measured via EAUS, and Wexner score was recorded using a questionnaire.

The Wexner continence score was used to assess the severity of fecal incontinence [18]. The scoring system comprised five items focused on the type and frequency of incontinence (solid, liquid, gas, and wearing a pad) and lifestyle alterations. Frequency was scored as follows: 0 = never; 1 = rarely (< 1/month); 2 = sometimes (< 1/week, ≥ 1/month); 3 = usually (< 1/day, ≥ 1/week); and 4 = always (≥ 1/day). The overall score is the sum of the scores of individual items; the lowest score (0) represents perfect continence, whereas the highest score (20) represents the worst incontinence.

IAS elasticity, anorectal function test values, and Wexner score were recorded 1–3 weeks before and 4–6 weeks after CRT. Thereafter, the relationship between elasticity before and after CRT was observed. Rectal surgery was typically performed 8–10 weeks after CRT (Online resource 1).

### Statistical analyses

Quantitative data are expressed as mean ± SD and scores are expressed as median and range. Chi-square and Mann–Whitney *U* tests were used to compare categorical and continuous variables, respectively. Spearman’s rank correlation coefficient was used for correlations regarding changes in IAS elasticity, MRP, and MSP. All statistical analyses were performed using JMP Pro 15.0.0 software (SAS Institute Inc., Cary, NC, USA). The level of significance was set at *p* < 0.05.

## Results

Table 1 presents the overall patient background. Preoperative CRT with an irinotecan-containing regimen was performed in 23/27 (85.2%) patients. A decrease in IAS elasticity (IAS sclerosis) was observed in 16/27 (59.3%) patients before and after preoperative CRT; the clinicopathological factors of this group were compared with those of the IAS non-sclerosis group (Table 2).

**Table 1:** Characteristics of the patients

**Variable**		**Total, *n* = 27**
Age (years)	Median (range)	63 (40–80)
Sex	Male/female	17/10
Tumor location from AV (cm)	Median (range)	5 (1–10)
Histology	Well/moderately differentiated/other	24/3
Clinical Stage	IIa/IIb/IIc/IIIa/IIIb/IIIc	8/0/1/3/10/5
Chemotherapy regimen	Tegafur-uracil, leucovorin + irinotecan/tegafur-uracil, leucovorin	23/4
Tumor regression grade	1/2/3	10/13/4
MRP (mmHg)		
Before CRT	Median (range)	58.0 (9.5–121.0)
After CRT	Median (range)	54.0 (16.5–112.0)
MSP (mmHg)		
Before CRT	Median (range)	168.5 (36.5–380.0)
After CRT	Median (range)	143.0 (66.0–382.0)
The IAS elasticity		
Before CRT	Median (range)	144.5 (24.9–249.4)
After CRT	Median (range)	110.4 (7.1–232.3)
Changes of the IAS elasticity before and after CRT	Increase / Decrease	11 / 16

Data are presented as number of cases. Continuous variables are expressed as median (range)

*AV* anal verge, *CRT*, chemoradiotherapy, *MRP* maximum resting pressure, *MSP* maximum squeeze pressure, *IAS* internal anal sphincter

**Table 2:** Clinicopathological characteristics of the patients

**Clinicopathological characteristic**	**Variable**	**Sclerosis (n = 16)**	**Non-sclerosis (n = 11)**	***p***
Age (years)	Median (range)	59.5 (40–80)	64 (57–72)	0.68
Sex	Male	8	9	0.09
BMI, kg/m^2^	Median (range)	22.6 (18.1–34.8)	22.8 (16.3–26.9)	0.94
Tumor location from AV (cm)	Median (range)	5.5 (1–8)	5 (2–10)	0.92
Histology	Well/moderately differentiated	14	10	0.78
Chemotherapy regimen	Tegafur-uracil, leucovorin + irinotecan	13	10	0.49
cT-stage	cT3/cT4	13/3	9/2	0.33
cN-stage	cN0/cN1-3	6/10	3/8	0.58
ypT-stage	ypT0-2/ypT3-4	4/12	9/2	0.004
ypN-stage	ypN0/ypN1-3	10/6	10/1	0.10
Tumor regression grade	1/2/3	9/6/1	1/7/3	0.01

Continuous variables are expressed as median (range)

*BMI* body mass index, *AV* anal verge

**Fig. 3:**
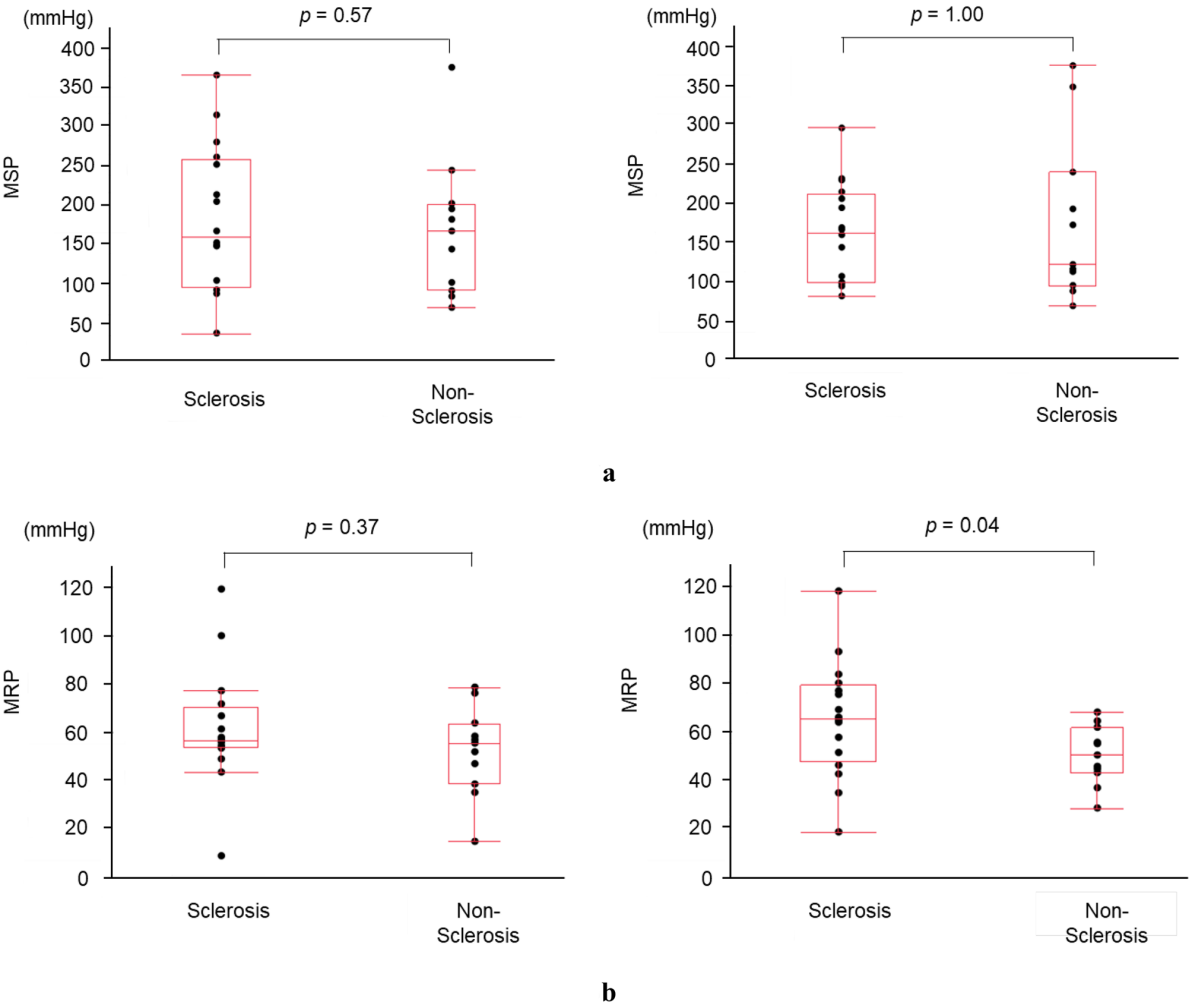
**a** Maximum resting pressure changes before and after chemoradiotherapy in the internal anal sphincter sclerosis and non-sclerosis groups; no significant difference was observed between the groups before chemoradiotherapy; however, a significant increase was observed in the maximum resting pressure in the sclerosis group after chemoradiotherapy. **b** Changes in the maximum squeeze pressure before and after chemoradiotherapy in the internal anal sphincter sclerosis and non-sclerosis groups; no significant difference was observed in maximum squeeze pressure changes before and after chemoradiotherapy between the groups

The sclerosis group had significantly higher ypT stage and lower tumor regression grade than the non-sclerosis group. Regarding anorectal manometry data, MRP after CRT was significantly higher in the IAS sclerosis group than in the non-sclerosis group (63.0 mmHg vs. 47.0 mmHg, *p* = 0.04); however, no significant difference was observed in MSP between the groups (163.0 mmHg vs. 120.5 mmHg, *p* = 1.00). (Fig. 3a, b).

**Fig. 4:**
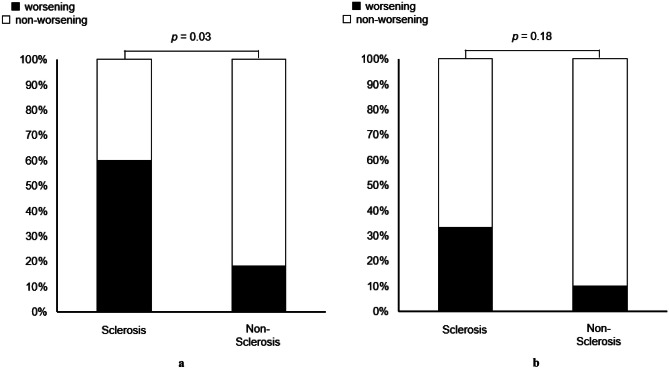
**a** Total Wexner score changes before and after chemoradiotherapy in the internal anal sphincter sclerosis and non-sclerosis groups. A significantly greater proportion of individuals in the internal anal sphincter sclerosis group had a worsening of their Wexner scores after chemoradiotherapy. **b** Analysis of gas incontinence on Wexner scores between the internal anal sphincter sclerosis and non-sclerosis groups. The internal anal sphincter sclerosis group had a higher percentage of worsening in the gas incontinence score; however, no significant differences were observed between the groups

Additionally, in the IAS sclerosis group, significantly more patients presented with a worsening total Wexner score than in the non-sclerosis group (60.0% vs. 18.2%, *p* = 0.03) (Fig. 4a). On analysis, the median Wexner score was significantly worse after CRT than before CRT in the sclerosis group (0 vs. 2, *p* = 0.03), while there was no difference in the Wexner score before and after CRT in the non-sclerosis group (4 vs. 0, *p* = 0.37) (Online resource 2). Gas incontinence worsened in the IAS sclerosis group (Fig. 4b); however, there was no significant difference between the groups’ gas incontinence and the other four parameters (solid incontinence, liquid incontinence, wearing of a pad, lifestyle alteration) (Online resource 3).

**Fig. 5:**
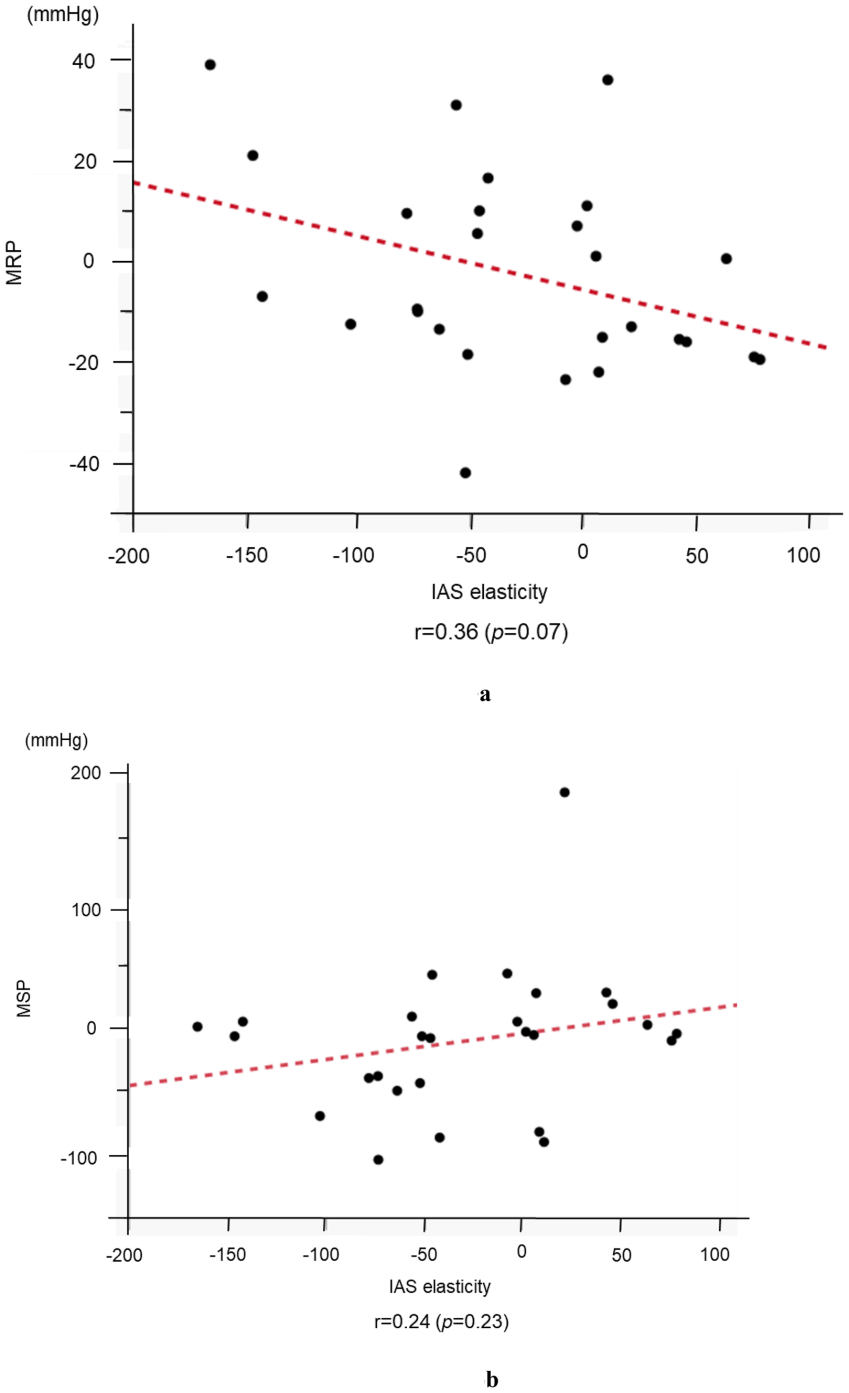
**a** Correlation between the changes in internal anal sphincter elasticity and maximum resting pressure before and after chemoradiotherapy. An inverse correlation was observed: the more the internal anal sphincter elasticity decreased, the more the internal anal sphincter sclerosis occurred and the more the maximum resting pressure increased. **b** Correlation between the changes in internal anal sphincter elasticity and maximum squeeze pressure before and after chemoradiotherapy. There was no obvious correlation between the changes in internal anal sphincter elasticity and maximum squeeze pressure

Finally, a comparison of the changes in IAS elasticity and those in MRP and MSP before and after preoperative CRT revealed that as elasticity decreased (IAS became harder), MRP increased (*r* = 0.36, *p* = 0.07). Conversely, no correlation was observed between the changes in elasticity and MSP (*r* = 0.24, *p* = 0.23) (Fig. 5a, b).

## Discussion

We compared patients with lower-advanced rectal cancer having IAS sclerosis and those without IAS sclerosis after preoperative CRT. We observed that the IAS sclerosis group had a significantly higher MRP and more patients with a worsening Wexner score than the non-sclerosis group. Additionally, we demonstrated that MRP increased as IAS elasticity decreased after preoperative CRT.

MRP reflects IAS function [19]; however, information on the physiological effects of CRT on anal function, including MRP, remains limited [20–22]. While a few studies have indicated that MRP decreases in the long term—1 year postoperatively [23–25]—Hirata et al. observed no significant difference in the MRP at 1 year postoperatively between the CRT-treated and non-CRT-treated groups [26]. In general, the long-term effects of preoperative CRT on postoperative anal function may vary owing to surgery-related factors such as postoperative complications (leakage, anastomotic height, and degree of nerve preservation). Moreover, a previous study demonstrated that the MRP is elevated in the short term, such as after CRT. Jang et al. compared 80 patients for whom pre-rectal manometry data were available before and after CRT and observed that the MRP elevated immediately after CRT [10]. Similarly, Iwamoto et al. reported a significant increase in the MRP immediately after completing definitive radiation therapy consisting of pelvic external and intracavitary irradiation in 16 patients with cervical cancer [20]. Based on previous reports, anal canal edema, enteric plexus damage, and anal sphincter fibrosis due to irradiation are possible reasons for the increase in the MRP after CRT [10, 20]. In this study, decreased elasticity was observed in 16/27 (59.3%) patients after CRT, indicating a moderate inverse correlation trend between IAS elasticity decrease and MRP increase. Da Silva et al. reported a higher percentage of fibrosis of the IAS in patients who underwent rectal resection after preoperative CRT [12]. If the elasticity decrease in the IAS reflects sclerosis due to increased fibrosis associated with CRT, IAS sclerosis may be responsible for the increase in MRP.

In our study, we observed a worsening Wexner score in the IAS sclerosis group after CRT compared with that in the non-sclerosis group. Similarly, Canda et al. and Lim et al. reported worsening Wexner scores due to irradiation in a short-term study of bowel function, such as immediately after preoperative CRT [11, 27]. Based on our study results, we infer that IAS sclerosis due to CRT may contribute to the worsening of the total Wexner score. In the examination of each item of the Wexner score for the IAS sclerosis and non-sclerosis groups, no significant difference was observed in the percentage of worsening in all parameters. However, the percentage of gas incontinence was higher in the sclerosis group than in the non-sclerosis group. This result might be attributed to the hardening of the IAS, which limited the physiological contraction of the IAS. Further analysis will be needed to clarify the cause of this difference.

The small number of patients limited a detailed study; however, in terms of patient background, the ypT stage was higher and TRG was lower in the IAS sclerosis group than in the non-sclerosis group. With regard to malignancy and fibrosis, cancer-associated fibroblasts (CAFs) are recognized to influence the progression and course of chemotherapy and radiation therapy in various types of tumors, including colorectal cancer (CRC). Therefore, the relationship between fibrosis around malignant tumors and therapeutic efficacy has received attention over recent years [28]. If there are clear indications that histological hardening and increased fibrosis cause changes in elasticity, the relationship between elasticity and the therapeutic effect of preoperative CRT could be examined and addressed in the future. This study focused on the correlation between elasticity and anorectal function before and after preoperative CRT. Therefore, the effect of IAS sclerosis and fibrosis on postoperative anorectal function is unknown and requires detailed investigations. Our findings suggest that real-time tissue elastography on IAS is a useful modality compared to anorectal function tests to predict the effect of preoperative CRT on anorectal function and quality of life because it can objectively evaluate the hardness of the IAS.

Our study has several limitations. First, as this was a single-institution study, the number of patients was small. Second, irinotecan administered preoperatively may have affected Wexner score after treatment. Third, the histological basis for IAS sclerosis as assessed by elasticity is unclear and should be studied in the future.

In conclusion, a decrease in IAS elasticity after CRT correlates with MRP increase and a worsening Wexner score. This finding suggests that CRT-induced IAS sclerosis may affect anorectal functions after CRT.

## Supplementary Information

Below is the link to the electronic supplementary material.

Supplementary file1 (TIF 1021 KB)

Supplementary file2 (PDF 294 KB)

Supplementary file3 (TIF 4708 KB)

Supplementary file4 (PDF 134 KB)

## Data Availability

The data that support the findings of this study are not openly available due to reasons of sensitivity and are available from the corresponding author upon reasonable request.
