# Supplementary figures and images for: Evaluation of anorectal function using real-time tissue elastography before and after preoperative chemoradiotherapy
Source: Int J Colorectal Dis. 2024 Apr 25;39(1):56. doi: 10.1007/s00384-024-04633-8 (PMC11045657; doi:10.1007/s00384-024-04633-8)

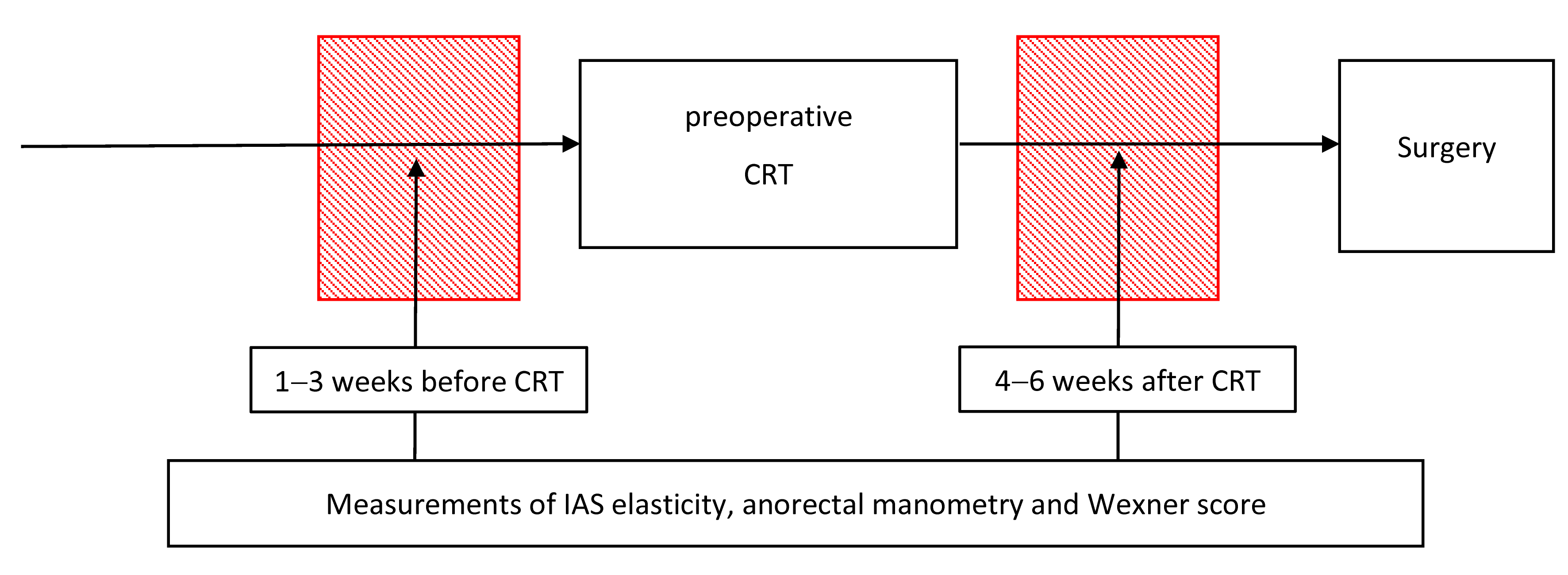

Supplement: Supplementary file 1 — Supplementary file1 (TIF 1021 KB) [file 384_2024_4633_MOESM1_ESM.tif]

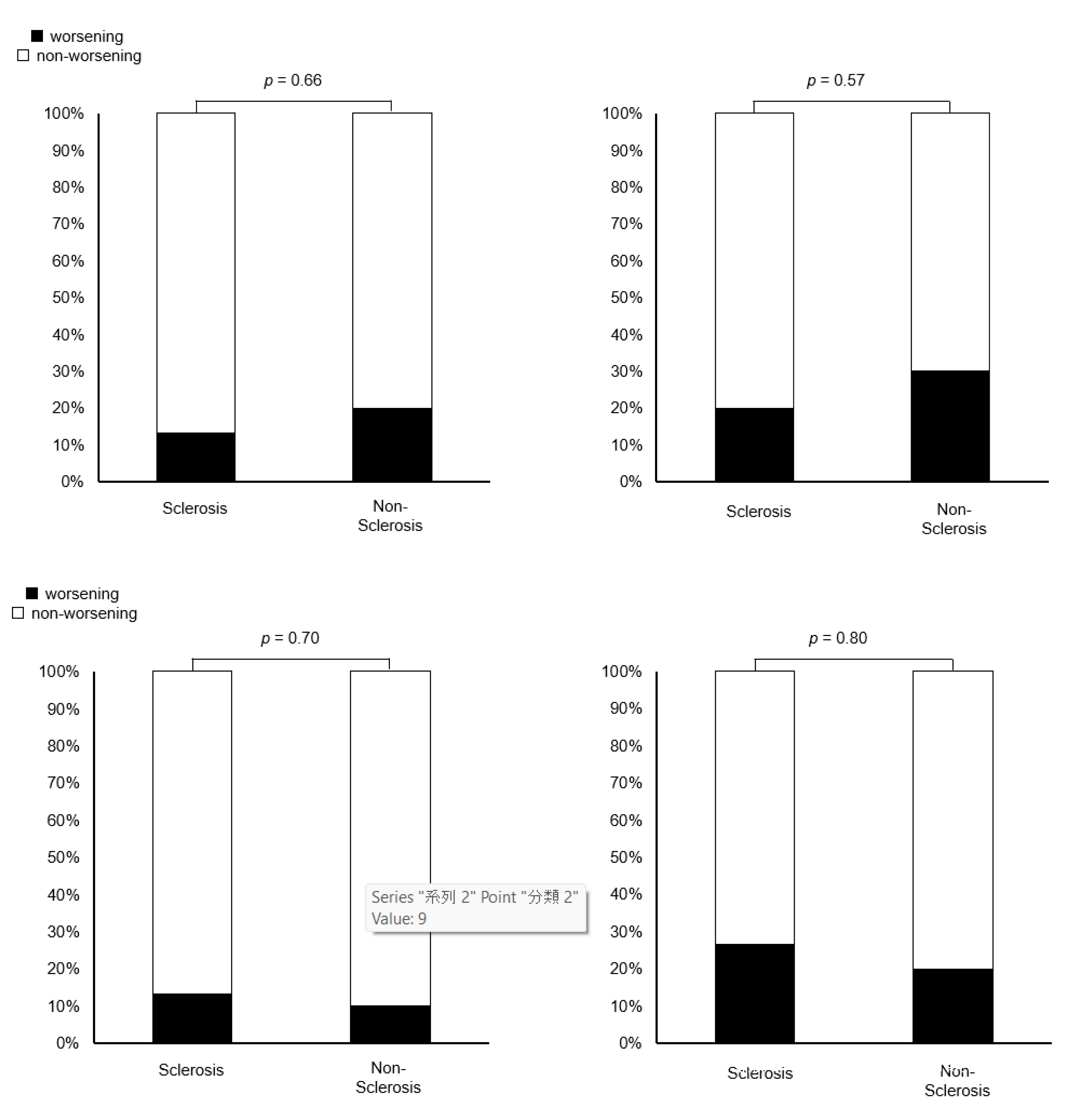

Supplement: Supplementary file 3 — Supplementary file3 (TIF 4708 KB) [file 384_2024_4633_MOESM3_ESM.tif]
