# Supplementary material for: Evaluation of anorectal function using real-time tissue elastography before and after preoperative chemoradiotherapy
Source: Int J Colorectal Dis. 2024 Apr 25;39(1):56. doi: 10.1007/s00384-024-04633-8 (PMC11045657; doi:10.1007/s00384-024-04633-8)
Supplement: Supplementary file 2 — Supplementary file2 (PDF 294 KB) [file 384_2024_4633_MOESM2_ESM.pdf]

Evaluation of anorectal function using real-time tissue elastography before and after preoperative chemoradiotherapy

Int J Colorectal Disease

Akira Sakamoto, M.D., Kazuhito Sasaki, M.D., Ph.D., Hiroaki Nozawa, M.D., Ph.D., Koji Murono, M.D., Ph.D., Shigenobu Emoto, M.D., Ph.D., Yuichiro Yokoyama, M.D., Ph.D., Hiroyuki Matsuzaki, M.D., Ph.D., Yuzo Nagai, M.D., Ph.D., Shinya Abe, M.D., Ph.D., Takahide Shinagawa, M.D., Ph.D., Hirofumi Sonoda, M.D., Ph.D., and Soichiro Ishihara M.D., Ph.D.

Department of Surgical Oncology, Faculty of Medicine, The University of Tokyo, Tokyo, Japan

Corresponding author:

Akira Sakamoto, M.D.

E-mail address: sakamotoa-sur@h.u-tokyo.ac.jp

Supplemental Table 1. Wexner score changes before and after CRT in the sclerosis and non-sclerosis groups

	Wexner score before CRT	Wexner score after CRT	*p*
Sclerosis	0 (0–9)	2 (0–9)	0.03
Non-sclerosis	4 (0-8)	0 (0-10)	0.38

CRT, chemoradiotherapy

Continuous variables are expressed as median (range).
